# Lymphoepithelial Subtype of Oral Squamous Cell Carcinoma: Report of an EBV-Negative Case and Literature Review

**DOI:** 10.3390/dj10090165

**Published:** 2022-09-05

**Authors:** Rodopi Emfietzoglou, Efstathios Pettas, Maria Georgaki, Erofili Papadopoulou, Vasileios Ionas Theofilou, Nikolaos Papadogeorgakis, Evangelia Piperi, Marcio Ajudarte Lopes, Nikolaos G. Nikitakis

**Affiliations:** 1 School of Dentistry, National and Kapodistrian University of Athens (NKUA), 11527 Athens, Greece; 2 Department of Oral Medicine & Pathology and Hospital Dentistry, School of Dentistry, National and Kapodistrian University of Athens (NKUA), 11527 Athens, Greece; 3 Department of Oncology and Diagnostic Sciences, School of Dentistry, University of Maryland, Baltimore, MD 21201, USA; 4 Department of Oral and Maxillofacial Surgery, School of Dentistry, National and Kapodistrian University of Athens (NKUA), 11527 Athens, Greece; 5 Department of Oral Diagnosis, Piracicaba Dental School, University of Campinas, Avenida Limeira 901, Piracicaba 13414-903, SP, Brazil

**Keywords:** lymphoepithelial carcinoma, squamous cell carcinoma, EBV, oral cavity, minor salivary glands, surface epithelium

## Abstract

Lymphoepithelial carcinoma (LEC) of the oral mucosa is a rare histopathologic subtype of squamous cell carcinoma (SCC), which shares morphologic similarities with nasopharyngeal carcinoma (NPC), non-keratinizing undifferentiated subtype. The admixture of neoplastic epithelial tumor cells and a dense lymphoplasmacytic infiltrate makes microscopic diagnosis challenging. LEC etiopathogenesis has been variably associated with Epstein–Barr virus (EBV) infection, depending on the specific anatomic location and racial predilection, with a higher incidence in endemic populations. Although described in several subsites of the head and neck region, including the major salivary glands, the oral mucosa is considered an infrequent location for LEC development, deriving either from minor salivary glands (MSGs) or the surface epithelium. Herein, we report a rare case of an EBV-negative LEC arising from the oral surface epithelium, presenting as gingival swelling, and review the pertinent English-language literature, which revealed only 26 previously reported oral LECs. Our case is only the fourth oral LEC originating from the surface epithelium and the first one to affect the gingiva.

## 1. Introduction

Lymphoepithelial carcinoma (LEC) represents a rare malignancy, described by the World Health Organization (WHO) as “a squamous cell carcinoma (SCC) morphologically similar to non-keratinizing nasopharyngeal carcinoma (NPC), undifferentiated subtype” [1]. When a diagnosis of LEC is considered, the evaluation of the nasopharynx is essential for the exclusion of a nasopharyngeal primary, as the separation of LEC from undifferentiated NPC is not microscopically feasible [2,3]. The expression of epithelial immunohistochemical markers by malignant cells helps rule out a wide spectrum of other neoplasms that may histopathologically mimic LEC [4]. This tumor may develop at several sites of the head and neck region, including the sinonasal tract, larynx, and oropharynx [1,5,6]; salivary glands also constitute a well-documented site of LEC development [4,7,8,9]. In endemic populations (South East Asians and Arctic region natives), where the incidence of NPC is also high, LEC is the most frequent type of salivary gland malignancy [10]. On the other hand, tumors arising from the surface squamous epithelium of the oral mucosa have rarely been described in the literature [5,11]. Herein, we report a rare case of an EBV-negative LEC presenting as gingival enlargement in a middle-aged man of Asian descent and comprehensively review the pertinent English-language literature focusing on the controversial origin of this tumor.

## 2. Case Report

A 51-year-old male of Filipino descent presented for evaluation of hemorrhagic swelling in the right mandibular region, first noticed one week before. The patient did not experience any general symptoms, such as discomfort, fever, night sweats, or weight loss. No previous history of tobacco and/or alcohol use was reported. His medical history was significant for hypertension, which was managed with amlodipine 10 mg/day for many years. Moreover, due to chronic kidney disease (present for the last 2 years), the patient was scheduled to begin hemodialysis in the following months.

**Figure 1:**
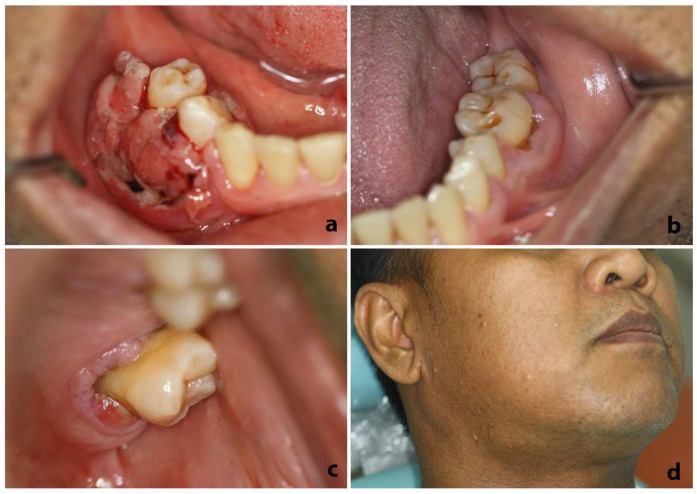
(**a**–**c**) Intraoral clinical examination showing (**a**) exophytic, hemorrhagic ulcerated mass surrounding the gingiva of the right mandibular premolars extending posteriorly to the edentulous alveolar mucosa, (**b**) gingival enlargement in the left posterior mandibular buccal gingiva, and (**c**) gingival enlargement in left posterior maxillary palatal gingiva. (**d**) Extraoral clinical examination demonstrating an enlarged lymph node in the right submandibular area.

Intraoral clinical examination revealed an exophytic, hemorrhagic, ulcerated mass of approximately 3 cm in maximum diameter surrounding the buccal and lingual gingiva of the right mandibular premolars, also extending to the edentulous alveolar mucosa posteriorly to the second premolar (Figure 1a). The affected teeth exhibited terminal mobility with approximately 1 cm pocket depth and bleeding on probing. Gingival enlargement was also observed in the left posterior mandibular buccal gingiva (Figure 1b), as well as the left posterior maxillary palatal gingiva (Figure 1c). Extraorally, a painful and almost fixed lymph node in the right submandibular area was palpated (Figure 1d).

**Figure 2:**
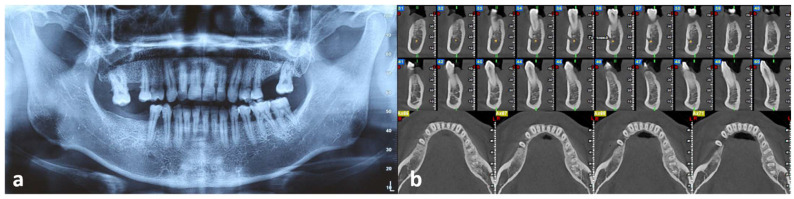
(**a**) Panoramic radiograph showing alveolar bone loss in proximity to the right mandibular premolar area. (**b**) Cross-sectional and axial views of cone-beam computed tomography (CBCT) revealing superficial resorption of the alveolar ridge and destruction of the buccal cortical bone in the area of the right mandibular premolars.

Panoramic radiograph showed alveolar bone loss in proximity to the right mandibular premolars (Figure 2a), while cone-beam computed tomography (CBCT) revealed superficial bone resorption of the alveolar ridge and the buccal cortex in an area measuring approximately 7 mm in length and extending approximately 3 mm in depth (Figure 2b).

The differential diagnosis (DD) of the right mandibular gingival growth mainly included various malignant neoplasms, such as hematologic malignancies (e.g., lymphoma) and sarcomas; carcinomas, such as SCC, could not be excluded, although the clinical appearance was not typical. Taking into account the presence of multifocal gingival lesions (which could be related to a common cause or not), other diagnostic considerations included diffuse gingival enlargement due to calcium channel blockers (with localized pyogenic granuloma-like growths) and hematologic diseases (such as leukemic infiltrate). Further, benign or reactive tumors with local aggressive behavior (such as peripheral giant cell granuloma or even brown tumor of secondary hyperparathyroidism induced by renal failure) were also considered.

**Figure 3:**
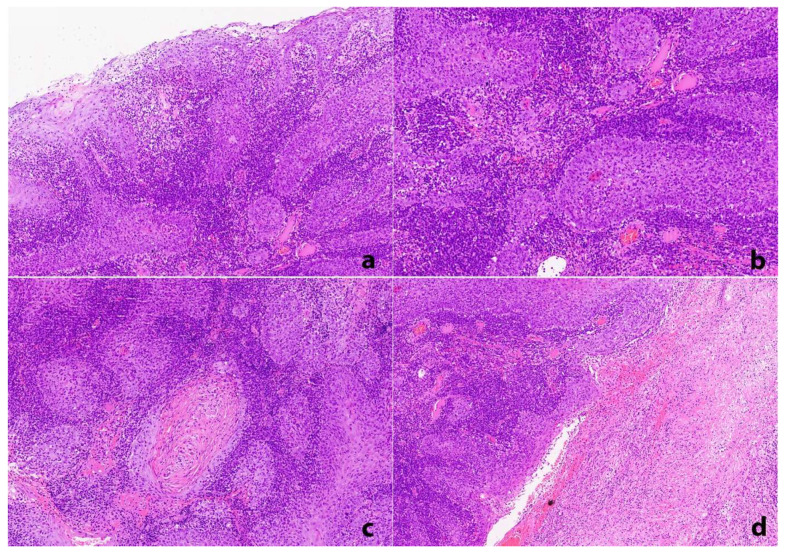
Histopathologic examination *(Hematoxylin and Eosin, initial magnification 200×)*: (**a**) diffuse infiltration of the underlying connective tissue by neoplastic cells arising from a partially ulcerated and non-keratinized stratified squamous epithelium, (**b**) diffuse dense lymphoplasmacytic cell infiltrate surrounding the neoplastic epithelial cells and obscuring the tumor islands, (**c**) focal keratin pearl formation, and (**d**) focal areas of tissue necrosis.

**Figure 4:**
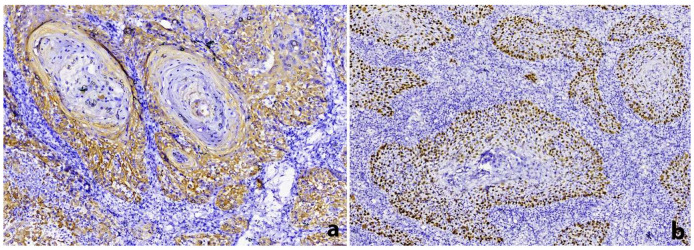
Immunohistochemical analysis showing positivity of the tumor cells for (**a**) cytokeratin AE1/AE3 and (**b**) p63.

Incisional biopsies under local anesthesia from all of the aforementioned gingival sites were performed. For the specimens taken from the left posterior mandibular buccal gingiva and left posterior maxillary palatal gingiva, typical features of hyperplastic gingivitis and pyogenic granuloma, respectively, were seen; for these lesions, a diagnosis of diffuse gingival enlargement, likely associated with the use of calcium channel blockers along with poor oral hygiene, was established. On the other hand, histopathologic examination of the right posterior mandibular buccal gingival specimen revealed diffuse infiltration of the connective tissue by neoplastic cells arising from the partially ulcerated oral mucosal surface epithelium (Figure 3a). The tumor cells were pleomorphic with sizeable hyperchromatic or vesicular nuclei and prominent eosinophilic nucleoli and were mostly arranged in syncytial islands; atypical mitotic figures and a high cell proliferation index were also noticed. A dense lymphoplasmacytic cell infiltrate was observed, diffusely and intimately surrounding the malignant epithelial cells and obscuring the tumor islands (Figure 3b); dispersed polymorphonuclear leukocytes and eosinophils were also present. Keratin pearl formation (Figure 3c) and areas of necrosis (Figure 3d) were focally observed. The neoplastic cells reacted positively to cytokeratin AE1/AE3 (Figure 4a), p63 (Figure 4b), CK5/6, and p40, confirming their squamous epithelial phenotype. The lack of p16 immunohistochemical staining and the absence of Human Papillomavirus (HPV)-16 nuclear expression by in situ hybridization (ISH) excluded the possibility of HPV involvement. LMP-1 immunohistochemistry and ISH for EBV encoding region (EBER) for small RNAs were also negative. A final diagnosis of oral LEC was rendered, and the patient was referred for staging and management.

Magnetic resonance imaging (MRI) of the head and neck confirmed the presence of a tumor infiltrating the mucosa of the right mandible measuring 3.2 × 1.0 × 0.8 cm and causing bone resorption in the premolar area; in addition, enlarged lymph nodes were seen in the right submandibular area, while no primary nasopharyngeal tumor was detected. Systemic evaluation via thorough clinical examination and imaging (PET/CT scan) was performed and showed no evidence of distant metastatic disease. Following the tumor board, the patient was managed by wide surgical resection with segmental mandibulectomy (from the second lateral incisor to the right mandibular angle with titanium plate reconstruction) and ipsilateral neck dissection. According to the histopathologic and immunohistochemical examination of the surgical specimen, the microscopic features were similar to those of the initial biopsy, confirming the diagnosis of LEC. The tumor size was 2.5 cm in maximum diameter, and the depth of invasion was estimated to be 5.2 mm; the margins were free of tumor, but 2 (out of 28) positive right cervical lymph nodes with a maximum diameter of 2 cm without extracapsular spread were detected; the tumor was classified as pT2N2bM0. The patient received adjuvant chemotherapy and radiation therapy. More specifically, the radiotherapy consisted of a total of 60 Gy (2 Gy per session, five sessions per week for 6 weeks) of VMAT-IMRT (Volumetric Modulated Arc Radiation Treatment—Intensity-Modulated Radiotherapy) in the right mandible and surrounding areas (right oropharynx, base of tongue, retropharyngeal lymph nodes, and cervical lymph node levels I and II); in addition, the patient received 54 Gy and 50 Gy in the right cervical lymph node levels III-V and left cervical lymph node levels II-V, respectively. The patient was placed on close follow-up; at 28 months post-treatment, he remains free of disease.

## 3. Discussion

A variety of terms have been used over the years to describe LEC in non-nasopharyngeal locations of the head and neck, such as lymphoepithelioma, lymphoepithelioma-like carcinoma, lymphoepithelial-like carcinoma, undifferentiated carcinoma of nasopharyngeal type, and undifferentiated carcinoma with lymphoid stroma, all of which highlight its histopathologic resemblance to its nasopharyngeal counterpart [11].

NPC is divided into three histopathologic variations: keratinizing, non-keratinizing, and basaloid SCC [2,3]. This tumor exhibits specific geographic distribution and frequently affects adult men from southern China (especially Hong Kong SAR) [2,3]. Non-keratinizing NPC, which resembles LEC, shows a strong association with EBV, especially in endemic populations [2,3]. At the time of diagnosis, advanced locoregional disease with the presence of cervical lymph node metastasis is the rule. Thus, for patients presenting with lymph node involvement by carcinoma of unknown primary origin, the exclusion of a nasopharyngeal primary is mandatory and could be performed by clinical, histopathologic, and endoscopic examination in combination with imaging techniques.

On microscopy, LEC displays a quite similar appearance to non-keratinizing NPC, undifferentiated subtype, with the ratio of the epithelial to lymphoid component varying from one tumor to another [3]. Similar to NPC, LEC should be tested for EBV; highly sensitive ISH for EBER is the most commonly used and reliable method for the detection of EBV, although PCR for EBV, less sensitive immunohistochemical LMP-1 reactivity, and non-specific EBV serology have also been employed [6,12]. However, molecular techniques provide the highest reliability, among which EBER ISH additionally enables specific localization of the viral genome within the affected cells [13]. With regard to LEC differential diagnosis, a wide spectrum of lesions, ranging from benign lymphoepithelial lesions to undifferentiated carcinomas, could be included, depending on the anatomic region involved [4,5]. For example, in the oral cavity (including minor salivary glands), lymphomas may exhibit microscopic features similar to those of LEC. Furthermore, few diffuse large B-cell lymphomas (DLBCLs) are EBV-associated [14]. Notwithstanding, the lack of an epithelial component in lymphomas, as confirmed by immunohistochemistry, will finally lead to the proper diagnosis.

Besides oral mucosa, LEC may be present in various areas of the head and neck region, i.e., nasal cavity and paranasal sinuses, larynx/hypopharynx/trachea/parapharyngeal space, oropharynx, and salivary glands.

Tumors of the sinonasal tract are rare, display a predilection for males and middle-aged to elderly patients (median age: 58 years), usually develop in the nasal cavity, and are commonly EBV-related (in >90% of cases) [1,5,15,16]. Obviously, the exclusion of an NPC primary spreading to the nearby sinonasal region is required. In the sinonasal tract, LEC should also be distinguished from sinonasal undifferentiated carcinoma (SNUC), which—in contrast to LEC—is commonly characterized by prominent apoptotic bodies and areas of necrotic tissue, while syncytial tumor islands are absent [5]. Sinonasal LEC has a 5-year survival rate of about 50% and shows a lower tendency of lymph node metastasis compared to NPC [1].

In the larynx/hypopharynx/trachea region, LEC is also rare, usually involves the larynx, infrequently involves the hypopharynx, and only rarely involves the trachea, shows a predilection for elderly men (mean age: 62 years), and affects mostly Caucasians [17]. Even though a relationship with EBV infection has been described [17], it is not as strong as in nasopharyngeal and sinonasal cases [1]. Regional lymph node metastasis occurs in approximately three-quarters of the cases, with a 5-year survival rate of approximately 60% [1].

In the oropharynx, among other SCC microscopic variations, the lymphoepithelial pattern is rarely diagnosed and usually characterizes p16-positive tumors [18]; in a study by Singhi et al. [6], all 22 cases of lymphoepithelial-like carcinoma of the oropharynx were p16-positive, 86% of them also exhibiting HPV positivity by ISH, while EBV was not detected in any studied case. Therefore, lymphoepithelial-like carcinomas of the head and neck, especially those presenting as cervical lymph node metastasis, should be tested for HPV, in addition to EBV, since they may derive from an HPV-positive lymphoepithelial SCC of oropharyngeal origin. To this end, p16 IHC followed by ISH for high-risk HPV subtypes (commonly -16 and -18) is the most reliable method for HPV investigation [19].

In salivary glands, LEC represents a well-documented, albeit unusual, malignancy, which is commonly EBV-related in endemic populations (such as Inuit and South East Asians), with the exception of Western citizens [4,7,8,9,10,20]. The vast majority of tumors affect the major salivary glands of elderly patients without gender predilection; parotid is the most common site of involvement [4,9]. Microscopic differential diagnosis may include other salivary gland lesions with a prominent lymphoid component, but with quite different biological behavior, such as lymphadenoma and Warthin’s tumor; however, these entities, besides being EBV-negative, usually show distinct microscopic features [4]. Interestingly, the presence of lymphoepithelial sialadenitis (LESA) in the LEC background—even focally—raises concerns about the possible co-existence of Sjogrens’s syndrome. In a series of 16 cases of salivary gland LEC, 81% of which displayed this feature, none of the patients was found to be positive for Sjogren’s syndrome laboratory testing [4]. It has been hypothesized that LESA associated with LEC represents a reactive phenomenon and not a precursor lesion [21]. Approximately 40% of patients with LEC of the salivary glands present with lymph node metastases, and the 5-year survival rate is estimated at 70–80%; importantly, individuals diagnosed with EBV-negative LEC of the major salivary glands may have better survival rates [4,9].

Oral LEC could derive either from the surface mucosal epithelium or from adjacent minor salivary glands (MSGs). In the present case, with the location of the tumor exclusively on the gingiva, where MSGs are absent (with the extremely rare exception of ectopic glandular tissue), the absence of MSGs in both the incisional biopsy and surgical specimen and, more importantly, the microscopic evidence of the origin of the invading cells from the overlying epithelium confirmed its origin from the mucosal surface.

A thorough review of the English-language literature on LECs of the oral cavity was conducted. Only tumors located in the oral cavity proper (i.e., the anterior border is considered the lip vermillion and includes the soft palate, but not the base of the tongue or the tonsils) were considered; in addition, lesions involving the major salivary glands were excluded. Only cases with sufficient details that fulfilled the WHO classification 2017 diagnostic criteria for LEC [1] were included. All LEC cases located in the oral cavity with an origin from either the MSG or the surface epithelium were encompassed.

The review of the pertinent literature yielded 27 cases (including our case), all of which were case reports or case series [5,11,22,23,24,25,26,27,28,29,30,31,32,33,34,35,36,37,38,39,40,41]. The available data are presented in Table 1 and summarized in Table 2. An origin from the MSG or surface mucosal epithelium was discussed in 17 cases, out of which 10 were reported to originate from MSGs, and 4 originated from the surface epithelium, while in 3 cases, a possible MSG derivation was supported. It is worth mentioning that, in the remaining cases, no reliable conclusions regarding the tissue of origin could be drawn, as the relationship of tumor cells with the surface epithelium or subjacent MSG was not clearly described. Therefore, our case is only the fourth oral LEC with a demonstrated origin from the surface mucosa and the first reported one to affect the gingiva; the other three cases originating from the surface epithelium were located in the lateral border of the tongue (two cases) [40,41] and the lower lip (one case) [11]. The remaining oral LEC cases (of MSG or unspecified origin) showed a predilection for the palate, followed by the lips (mostly the lower) and the buccal mucosa; interestingly, two cases showed intrabony involvement (one in the maxilla and one in the mandible) [30,36], raising the possibility of an origin from odontogenic epithelial rests or entrapped MSG in the jaws (similar to other salivary gland neoplasms developing in the jaws) [42].

Oral LEC displays a slight female predilection, with a 1.25:1 female-to-male ratio. The majority of patients were Asians, particularly Chinese, with only six Caucasian patients reported so far; age ranged from 11 to 82 years with a mean age of 56.6 years. Regarding cases originating from the surface epithelium, two were Japanese, one was Caucasian, and one was Filipino, with a mean age of 69.5 years. Among cases with sufficient clinical details, most patients (14/24) presented with a lump, mass, or swelling, followed by an ulcer (6/24) or an ulcerated mass (4/24); pain was reported in 6 out of 13 cases with available information on symptomatology. The average duration before seeking diagnosis was 7.9 months, ranging from 1 week (in our case) to 36 months.

EBV involvement was investigated in 23 cases, among which 12 were reported as positive and 11 were negative; however, the most reliable molecular investigation, mainly via EBER ISH (or more rarely PCR), was performed in 11 positive and 9 negative cases. Notably, in some cases, the EBV serology test [27] or LMP-1 IHC [29] was negative, while EBV presence was finally detected through EBER ISH, highlighting its higher sensitivity. Moreover, an important observation was the lack of EBV (tested through molecular techniques) in all four cases originating from the surface mucosa; further reporting of such cases and their association or lack thereof with EBV infection is warranted.

Given the microscopic similarity between LEC and NPC, exclusion of a nasopharyngeal primary may be considered mandatory; in our review, the nasopharyngeal area was indeed evaluated in the majority (18/27) of cases by means of endoscopy with or without biopsy and/or imaging, with no primary tumor to be observed.

Lymph node metastasis was reported in almost half (12/26, 46.2%) of oral LEC cases with sufficient information. This is similar to LECs of the major salivary glands, which also have a tendency for regional metastatic spread to cervical lymph nodes, ranging from 40.5% to 45% among the series, mandating elective neck dissection, although the impact of lymph node involvement at presentation on the patient’s outcome remains questionable [43,44,45]. Among the four oral cavity LECs with an origin from the oral surface epithelium, only our patient developed ipsilateral lymph node involvement, classified as N2b according to the TNM staging system; however, the limited number of cases precludes definitive conclusions.

Surgical excision alone or in combination with adjuvant radiation therapy and, on certain occasions, chemotherapy was the most commonly used therapeutic intervention. In general, non-nasopharyngeal LEC of the head and neck, including oral and oropharyngeal tumors, are considered radiosensitive cancers with high rates of locoregional control [1,21,37,46]. The potential application of novel targeted therapies deserves further investigation; for example, c-kit (CD117) overexpression in an oral LEC case may suggest a possible role of imatinib (tyrosine kinase inhibitor) in the treatment of a subset of these tumors [29].

Follow-up data could be evaluated in 22 out of 27 patients, with an overall mean follow-up of 30.2 months (range: 7–120 months). Notably, the majority of patients (19/22, 86.4%) were alive with no evidence of disease, while only 2 patients (9.1%) died of disease; finally, one patient (4.5%), who received chemo- and radiation therapy with no surgical intervention, was alive with disease [24]. In general, oral LEC is considered to have a better prognosis in comparison to its nasopharyngeal counterpart, possibly because the oral cavity is more easily accessible than the nasopharynx, where prominent lymphatic drainage may lead to early dissemination of the disease [27]. Despite the limited number of patients with oral LEC of surface epithelium origin, all four of them (including ours) were alive without disease during a follow-up period ranging from 7 to 28 months.

## 4. Conclusions

LEC of the oral cavity is an uncommon subtype of SCC, which bears histopathologic resemblance to non-keratinizing undifferentiated NPC and, due to its rarity, along with the microscopic blending of neoplastic epithelial and non-neoplastic lymphoid cells, may cause diagnostic difficulties. When affecting the oral mucosa, LEC may derive from either the MSG or, ostensibly more rarely, from the surface epithelium. Exclusion of an NPC primary and the molecular evaluation of EBV involvement are required.

## Figures and Tables

**Table 1:** Summary of published LEC cases of the oral cavity proper in English-language literature.

Authors (year)	Gender	Race	Age	Clinical Signs and Symptoms	Duration (Months)	Site of Involvement	Tissue of Origin	EBV Investigation	Evaluation of Nasopharyngeal Area	Lymph Node Metastasis	Treatment	Follow- Up/ Outcome
Sadoff and Eckberg [22] (1973)	Female	NA	16	Painless lump	NA	Soft palate	NS	NA	NA	No	Surgery and radiation therapy	NS; NED
Weiss et al. [23] (1989)	Male	Caucasian	64	NA	NA	Floor of mouth	NS	EBER ISH: −	NA	NA *^1^	NA	NA
Evans and Guthrie [24] (1991)	Female	Caucasian	68	Ulcer	1.25	Soft palate and uvula	MSG	NA	Endoscopy	Yes	Radiation therapy	NS; NED
Worley and Daroca [25] (1997)	Female	Caucasian	69	Firm mass	3	Buccal mucosa	MSG	LMP-1 IHC: −	Endoscopy	Yes	Surgery and radiation therapy	12 months; NED
Ahuja et al. [26] (1999) *^2^	Female	Chinese	66	NA	NA	Soft and hard palate	MSG	EBER ISH: +	Endoscopy, biopsy, CT scan, MRI	Yes	NA	NA
	Male	Chinese	63	Slowly growing, painless ulcerated mass	36	Hard palate	MSG	EBER ISH: +	Endoscopy, biopsy, CT scan, MRI	No	NA	NA
	Female	Chinese	47	Mass, difficulty in swallowing, throat pain	NA	Soft palate and uvula	MSG	EBER ISH: +	MRI, biopsy	Yes	NA	NA
Chow et al. [27] (2002)	Male	Chinese	58	Painful ulcer	18	Junction of hard and soft palate	NS	EBER ISH: +, serology: −	Endoscopy, biopsy	Yes	Radiation therapy	30 months; NED
	Female	Chinese	56	Painless lump	12	Soft palate	Possibly MSG	EBER ISH: +, serology: −	NA	No	Radiation therapy	12 months; NED
	Female	Chinese	80	Ulcerative mass	1	Retromolar region	NS	EBER ISH: +	NA	Yes	No treatment	34 months; DOD
Hsiung et al. [28] (2005)	Female	NA	50	NA	NA	Buccal mucosa	MSG	NA	NA	No	Surgery and radiation therapy	116.5 months; NED
Lu et al. [29] (2006)	Female	Taiwanese	50	Painless firm mass	12	Buccal mucosa	MSG	EBER ISH: +, LMP-1 IHC: −	CT scan	No	Surgery and radiation therapy	120 months; NED
Mahomed and Grayson [11] (2008)	Male	Caucasian	73	Ulcer with irregular raised borders	4	Lower lip (vermillion- mucosa junction)	Surface epithelium	LMP-1 IHC: −, EBV PCR: −	NA	No	Surgery	20 months; NED
Shet et al. [30] (2009)	Male	NA	11	Diffuse jaw swelling	3	Mandible	Possibly MSG	EBER ISH: +	MRI	Yes	Surgery, chemotherapy, and radiation therapy	36 months; NED
Hsieh et al. [31] (2010)	Female	Taiwanese	50	Painless firm mass	3	Buccal mucosa	Possibly MSG	EBER ISH: +, serology: +	Biopsy, CT scan, Ga-67 whole body scan	No	Chemotherapy and radiation therapy	18 months; AWD
Rytkonen et al. [5] (2011)	Male	NA	49	Solid mass	6	Soft palate and uvula	NS	EBER ISH: −, IHC: −	Endoscopy	Yes	Surgery, chemotherapy, and radiation therapy	10 months; NED
Menditti et al. [32] (2012)	Male	Caucasian	56	Painless mass	2	Upper lip	MSG	Serology: −, LMP-1 IHC: −	CT scan	No	Surgery	24 months; NED
Ban et al. [33] (2014)	Male	Chinese	38	Mass	8	Hard palate	MSG	EBER ISH: (most likely) + *^3^	Endoscopy, CT scan	Yes	NA	NA
Gultekin et al. [34] (2014)	Male	NA	41	Non-healing ulcer	NA	Lower lip	NS	IHC: -, EBV PCR: −	PET/CT scan	Yes	Surgery, radiation therapy, and chemotherapy	36 months; NED
Zeng et al. [35] (2015)	Female	Chinese	38	Painless nodular soft mass	1	Hard palate	MSG	EBER ISH: +	Endoscopy, biopsy, MRI	No	Surgery	12 months; NED
Kamboj et al. [36] (2016)	Female	Indian	45	Painful swelling, nasal discharge	4	Maxilla	NS	NA	NA	No	Surgery	24 months; NED
Almeida et al. [37] (2020)	Female	Caucasian	82	Painful ulceration	12	Lower lip	NS	EBER ISH: −	NA	No	Surgery	24 months; NED
Shimizu et al. [38] (2017)	Male	Japanese	82	Hemorrhagic ulcerative indurated swelling	3	Floor of mouth	NS	EBER ISH: −	CT scan, PET/CT scan	Yes	Palliative therapy	12 months; DOD
Sayad et al. [39] (2021)	Female	NA	70	Painful ulceration	12	Lower lip (vermillion border)	NS	Serology: +	NA	No	Surgery and radiation therapy	16 months; NED
Takeda et al. [40] (2021)	Female	Japanese	72	Exophytic indurated mass (first presented as white patch)	16	Tongue (lateral border)	Surface epithelium	EBER ISH: −	CT scan, PET/CT scan	No	Surgery	12 months; NED
Ono et al. [41] (2021)	Male	Japanese	82	Hard mass	NA	Tongue (posterior-lateral border)	Surface epithelium	EBER ISH: −	Endoscopy, CT scan	No	Surgery	7 months; NED
Present case (2022)	Male	Filipino	51	Painful exophytic hemorrhagic ulcerated mass	0.25	Mandibular gingiva	Surface epithelium	EBER ISH: −, LMP-1 IHC: −	CT scan, MRI	Yes	Surgery, radiation therapy, and chemotherapy	28 month; NED

Abbreviations: MSG: minor salivary glands; NS: not specified; NA: not available; NED: no evidence of disease; DOD: died of disease; AWD: alive with disease. *^1^ The authors mention that a metastatic lesion was examined, but the location of metastasis is not specified. *^2^ The authors included four cases of LEC involving the palate; however, the fourth case is excluded, because it arose from the floor of the nasal cavity extending to the palate. *^3^ This case is part of a series of 28 LEC of salivary glands, including one case of MSG of the palate. ISH EBER positivity was detected in 27 out of 28 cases (without specifying the location of the 1 negative case)

**Table 2:** Summary of data of published cases of LEC in oral cavity proper.

Characteristics	Number
**Cases**	27
**Gender**	
Female	15
Male	12
**Race**	
Chinese	8
Caucasian	6
Japanese	3
Taiwanese	2
Indian	1
Filipino	1
*Not available*	*6*
**Age (years)**	
Range	11–82
Mean	56.6
Median	56
**Clinical signs**	
Lump/mass/swelling	14
Ulcer	6
Ulcerated mass	4
*Not available*	*3*
**Symptoms**	
Painful	6
Painless	7
*Not available*	*14*
**Duration (months)** *(available in 20 cases)*	
Range	0.25–36
Mean	7.9
Median	4
**Site of involvement**	
Palate	10
*Soft*	*5*
*Hard*	*3*
*Both*	*2*
Lips	5
*Lower lip*	*4*
*Upper lip*	*1*
Buccal mucosa	4
Jaws	2
*Maxilla*	*1*
*Mandible*	*1*
Tongue	2
Floor of mouth	2
Retromolar region	1
Gingiva	1
**Origin**	
MSG	10
Possibly MSG	3
Surface epithelium	4
*Not specified*	*10*
**EBV investigation**	
Positive	12
*ISH*	*11*
*Other (serology)*	*1*
Negative	11
*ISH or PCR+IHC*	*9*
*Other (IHC +/-serology)*	*2*
*Not available*	4
**Lymph node metastasis**	
Yes	12
No	14
*Not available*	1
**Length of follow-up (months)** *(available in 20 cases)*	
Range	7–120
Mean	30.2
Median	22
**Patient outcome**	
No evidence of disease	19
Died of disease	2
Alive with disease	1
*Not available*	*5*

## Data Availability

Department of Oral Medicine & Pathology and Hospital Dentistry.

## References

[1] Bishop J.A., Gaulard P., Gillison M., El-Naggar A.K., Chan J.K.C., Grandis J.R., Takata T., Slootweg P.J. (2017). Lymphoepithelial carcinoma. WHO Classification of Head and Neck Tumors.

[2] Thompson L.D.R. (2007). Update on nasopharyngeal carcinoma. Head Neck Pathol..

[3] Petersson B.F., Belli D., El-Mofty S.K., Gillison M., Lewis J.S., Nadal A., Nicolai A., Wenig B.M., El-Naggar A.K., Chan J.K.C., Grandis J.R., Takata T., Slootweg P.J. (2017). Nasopharyngeal carci-noma. WHO Classification of Head and Neck Tumors.

[4] Whaley R.D., Carlos R., Bishop J.A., Rooper L., Thompson L.D.R. (2020). Lymphoepithelial carcinoma of salivary gland EBV-association in endemic versus non-endemic patients: A report of 16 cases. Head Neck Pathol..

[5] Rytkönen A.E., Hirvikoski P.P., Salo T.A. (2011). Lymphoepithelial carcinoma: Two case reports and a systematic review of oral and sinonasal cases. Head Neck Pathol..

[6] Singhi A.D., Stelow E.B., Mills S.E., Westra W.H. (2010). Lymphoepithelial-like carcinoma of the oropharynx: A morphologic variant of HPV-related head and neck carcinoma. Am. J. Surg. Pathol..

[7] Li L.-J., Li Y., Wen Y.-M., Liu H., Zhao H.-W. (2008). Clinical analysis of salivary gland tumor cases in west China in past 50 years. Oral Oncol..

[8] Wang Y.-L., Zhu Y.-X., Chen T.-Z., Wang Y., Sun G.-H., Zhang L., Huang C.-P., Wang Z.-Y., Shen Q., Li D.-S. (2012). Clinicopathologic study of 1176 salivary gland tumors in a Chinese population: Experience of one cancer center 1997–2007. Acta Oto Laryngol..

[9] Lewis J.S., El-Mofty S.K., Nicolai P., El-Naggar A.K., Chan J.K.C., Grandis J.R., Takata T., Slootweg P.J. (2017). Lymphoepithelial carcinoma. WHO Classification of Head and Neck Tumors.

[10] Krishnamurthy S., Lanier A.P., Dohan P., Lanier J.F., Henle W. (1987). Salivary gland cancer in Alaskan natives, 1966–1980. Hum. Pathol..

[11] Mahomed F., Grayson W. (2008). A rare case of lymphoepithelial carcinoma of the lip. Oral Surg. Oral Med. Oral Pathol. Oral Radiol. Endodontol..

[12] Neel H.B., Pearson G.R., Weiland L.H., Taylor W.F., Goepfert H.H., Pilch B.Z., Goodman M., Lanier A.P., Huang A.T., Hyams V.J. (1983). Application of Epstein-Barr virus serology to the diagnosis and staging of north American patients with nasopharyngeal carcinoma. Otolaryngol. Head Neck Surg..

[13] Gulley M.L. (2001). Molecular diagnosis of Epstein-Barr virus-related diseases. J. Mol. Diagn..

[14] Rodrigues-Fernandes C.I., Junior A.G., Soares C.D., Morais T.M.D.L., Amaral-Silva G.K.D., de Carvalho M.G.F., de Souza L.L., Pires F.R., dos Santos T.C.R.B., Pereira D.L. (2020). Oral and oropharyngeal diffuse large B-cell lymphoma and high-grade B-cell lymphoma: A clinicopathologic and prognostic study of 69 cases. Oral Surg. Oral Med. Oral Pathol. Oral Radiol..

[15] Thompson L.D. (2006). Sinonasal carcinomas. Curr. Diagn. Pathol..

[16] Zong Y., Liu K., Zhong B., Chen G., Wu W. (2001). Epstein-Barr virus infection of sinonasal lymphoepithelial carcinoma in Guangzhou. Chin. Med. J..

[17] Thompson L.D. (2017). Laryngeal dysplasia, squamous cell carcinoma, and variants. Surg. Pathol. Clin..

[18] Thompson L.D.R., Burchette R., Iganej S., Bhattasali O. (2020). Oropharyngeal squamous cell carcinoma in 390 patients: Analysis of clinical and histological criteria which significantly impact outcome. Head Neck Pathol..

[19] Carpenter D.H., El-Mofty S.K., Lewis J.S. (2011). Undifferentiated carcinoma of the oropharynx: A human papilloma-virus-associated tumor with a favorable prognosis. Mod. Pathol..

[20] Jones A.V., Craig G.T., Speight P.M., Franklin C.D. (2008). The range and demographics of salivary gland tumours diagnosed in a UK population. Oral Oncol..

[21] Wenig B.M. (2015). Lymphoepithelial-like carcinomas of the head and neck. Semin. Diagn. Pathol..

[22] Sadoff L., Eckberg T. (1973). Lymphoepithelioma after long-term tetracycline for acne. Lancet.

[23] Weiss L.M., Movahed L.A., Butler A.E., Swanson S.A., Frierson H.F., Cooper P.H., Colby T.V., Mills S.E. (1989). Analysis of lymphoepithelioma and lymphoepithelioma-like carcinomas for Epstein-Barr viral genomes by in situ hybridization. Am. J. Surg. Pathol..

[24] Evans A.T., Guthrie W. (1991). Lymphoepithelioma-like carcinoma of the uvula and soft palate: A rare lesion in an unusual site. Histopathology.

[25] Worley N.K., Daroca P.J. (1997). Lymphoepithelial carcinoma of the minor salivary gland. Arch. Otolaryngol. Head Neck Surg..

[26] Ahuja A.T., Teo P.M., To K.F., King A.D., Metreweli C. (1999). Palatal lymphoepitheliomas and a review of head and neck lymphoepitheliomas. Clin. Radiol..

[27] Chow T.L., Chow T.K., Lui Y.H., Sze W.M., Yuen N.W.F., Kwok S.P.Y. (2002). Lymphoepithelioma-like carcinoma of oral cavity: Report of three cases and literature review. Int. J. Oral Maxillofac. Surg..

[28] Hsiung C.-Y., Huang C.-C., Wang C.-J., Huang E.-Y., Huang H.-Y. (2006). Lymphoepithelioma-like carcinoma of salivary glands: Treatment results and failure patterns. Br. J. Radiol..

[29] Lu S.-Y., Huang C.-C., Hsiung C.-Y., Eng H.-L., Huang H.-Y. (2006). Primary lymphoepithelioma-like carcinoma of minor salivary gland: A case report with immunohistochemical and in situ hybridization studies. Head Neck.

[30] Shet T., Arora B., Laskar S., Basak R., Kane S., Kurkure P. (2009). Epstein-Barr virus–associated lymphoepithelioma-like carcinoma of mandible. Pediatr. Dev. Pathol..

[31] Hsieh M.Y., Chen Y.K., Lin L.M. (2010). Primary buccal lymphoepithelial carcinoma: Report of a case. Cases J. TAOMFR.

[32] Menditti D., Laino L., Milano M., Caputo C., Boccellino M., D’Avino A., Baldi A. (2012). Intraoral lymphoepithelial carcinoma of the minor salivary glands. Vivo.

[33] Ban X., Wu J., Mo Y., Yang Q., Liu X., Xie C., Zhang R. (2014). Lymphoepithelial carcinoma of the salivary gland: Morphologic patterns and imaging features on CT and MRI. AJNR Am. J. Neuroradiol..

[34] Gultekin M., Sari S.Y., Gunhan O., Hosal S., Cengiz M., Gurkaynak M. (2014). Lymphoepithelial carcinoma of the lower lip: Report of a case. Int. J. Hematol. Oncol..

[35] Zeng M., Li S., Fu J., Wu H., Gao Y. (2014). Primary lymphoepithelial carcinoma of the intraoral minor salivary gland: A case report. Oncol. Lett..

[36] Kamboj M., Chaturvedi M., Shreedhar B. (2016). Lymphoepithelioma-like carcinoma of the oral cavity-a diagnostic perplexion. Dent. J. Adv. Stud..

[37] Almeida L.Y., Silveira H.A., Silva E.V., Barbeiro C.D.O., De Paula J.A., Bufalino A., Ribeiro-Silva A., León J.E. (2019). EBV-negative lymphoepithelial-like carcinoma of the lower lip. Autops. Case Rep..

[38] Shimizu S., Miyazaki A., Nakamori K., Nakai H., Ogi K., Hasegawa T., Hiratsuka H. (2017). Immunophenotypic analysis of tumor infiltrating lymphocytes in Epstein-Barr virus-negative lymphoepithelial carcinoma of the oral cavity: Report of a case. J. Oral Maxillofac. Surg. Med. Pathol..

[39] Sayad Z., Hamidi O., Elouazzani H., Benazzou S., Cherradi N., Boulaadas M. (2021). Lymphoepithelioma-like carcinoma of the vermilion of lower lip: A case report and review of literature. Int. J. Innov..

[40] Takeda D., Shigeoka M., Sugano T., Yatagai N., Hasegawa T., Akashi M. (2021). A case report of tongue lymphoepithelial carcinoma with a histological diagnostic dilemma. Diagn..

[41] Ono S., Marunaka H., Yanai H., Kawai H., Takabatake K., Nishida K., Toji T., Nakano K., Nagatsuka H., Yoshino T. (2021). Lymphoepithelial carcinoma in the lateral tongue: The case report. Reports.

[42] Bruner J.M., Batsakis J.G. (1991). Salivary neoplasms of the jaw bones with particular reference to central mucoepidermoid carcinomas. Ann. Otol. Rhinol. Laryngol..

[43] Li F., Zhu G., Wang Y., Wang Y., Chen T., Ji Q. (2014). A clinical analysis of 37 cases with lymphoepithelial carcinoma of the major salivary gland treated by surgical resection and postoperative radiotherapy: A single institution study. Med. Oncol..

[44] Wang C.P., Chang Y.L., Ko J.Y., Lou P.J., Yeh C.F., Sheen T.-S. (2004). Lymphoepithelial carcinoma versus large cell undiferentiated carcinoma of the major salivary glands. Cancer.

[45] Zhan K.Y., Nicolli E.A., Khaja S.F., Day T.A. (2015). Lymphoepithelial carcinoma of the major salivary glands: Predictors of survival in a non-endemic region. Oral Oncol..

[46] Dubey P., Ha C.S., Ang K.K., El-Naggar A.K., Knapp C., Byers R.M., Morrison W.H. (1998). Nonnasopharyngeal lymphoepithelioma of the head and neck. Cancer.

